# Impact of Whole Legume Flour Addition on the Physicochemical, Textural, and Sensory Properties of Greek Fermented Flatbread “Langites”

**DOI:** 10.1155/ijfo/5816153

**Published:** 2026-10-22

**Authors:** Andriana E. Lazou, Efstathios D. Gkikas, Dimitra-Markella Brakoumatsou, Eris-Marina Tsompanidou, Styliani Protonotariou

**Affiliations:** ^1^ Laboratory of Chemistry Analysis and Design of Food Processes Department of Food Science and Technology School of Food Sciences University of West Attica Athens Greece uniwa.gr

**Keywords:** flatbread, image analysis, sensory evaluation, texture

## Abstract

Langites, a traditional flatbread made from a fermented batter baked at 210°C, was investigated regarding the effects of replacing wheat flour with whole‐grain red lentil or chickpea flour on the physicochemical, structural, textural, and sensory properties of langites. Wheat flour was substituted with each legume flour at 0%, 10%, and 30%, and flour functionality and product quality were comprehensively evaluated. Both legume flours exhibited substantially coarser particle‐size distributions than wheat flour, with median particle sizes (D50) of 321.2 *μ*m for chickpea, compared with 122.5 *μ*m for wheat flour. The incorporation of legume flours increased water absorption capacity, reaching 2.56 g/g for chickpea blends and 2.40 g/g for lentil blends, whereas higher water solubility was observed in lentil formulations (4.32%). Flour and product color changed significantly, with color differences (*Δ* *E*) reaching 23.57 for red lentil and 10.93 for chickpea flour. In langites, increasing legume substitution significantly increased moisture content, hardness, gumminess, and chewiness, whereas cohesiveness decreased from 0.85 to 0.80 in the 30% lentil formulation. Image analysis revealed a marked reduction in the number and uniformity of surface eyes, accompanied by an increase in mean eye diameter from 0.97 mm (control) to 1.29 mm and 1.14 mm in langites containing 30% lentil and chickpea flour, respectively, indicating impaired gluten development and reduced gas retention. Sensory evaluation showed that 10% substitution received good acceptability (7.26–7.59) compared with the wheat control (8.21), whereas 30% substitution significantly reduced acceptance, particularly for lentil flour (4.19). Principal component analysis demonstrated that overall acceptability was positively associated with eye number and sweet‐taste perception and negatively associated with hardness, chewiness, larger irregular pores, and color intensity. Overall, the 10% substitution with whole‐grain legume flour represents a promising formulation with enhanced nutritional value while preserving desirable technological and sensory characteristics.

## 1. Introduction

Globally, various types of flatbreads exist, with many processing technologies either locally developed or in the experimental phase, necessitating further research for industrial production. The future success of these products relies on consumer acceptance, influenced by tourism, gastronomic trends, and cuisine culture, potentially transforming regional businesses into international markets [1].

Under the name “flatbreads,” a wide variety of thin (several mm to several cm) baked products, which are produced worldwide, is classified. Although originated in the Mediterranean, Caucasian region, Arabian Peninsula, and Indian subcontinent, flatbreads are also produced in Central and South America [2–4]. Further, it should be noted that flatbreads increase food system sustainability by using local cereals, requiring short baking times, reducing tableware and water consumption, being easy to transport, and showing a variety of shelf lives, which may become long when baked to dryness [3].

As stated by Pasqualone [5], flatbreads can be classified by considering dough consistency, leavening, layered, or nonlayered structure of the finished product, and baking mode. Among them, a great category is occupied by flatbreads produced using semifluid batters onto cooking plates after fermentation. Yeast‐raised, fermented flatbreads are flat, leavened breads or crepes that undergo a fermentation process using baker′s yeast or sourdough to create a lighter, softer texture, and a distinct flavor. In Greece, and especially in the Thrace region, a kind of flatbread, having the form of a crepe, is prepared and consumed under the name “langites” or “lalangites” (singular: langita, plural: langites; the type almost entirely used). It is a yeast‐fermented product prepared using wheat flour, water, a little salt, and baker′s yeast and/or locally prepared sourdough. Sometimes eggs, sugar, a little milk, baking powder, and baking soda are added, the quantity of which depends on the local recipe. During preparation, yeast and salt are dissolved in lukewarm water, and then the wheat flour is added in small doses under stirring and beating until an easy flowing, rather thick batter is obtained. The dough is then left to ferment in a warm place until it rises and its surface fills with bubbles. The dough is baked by pouring and spreading it on a hot, flat, round, and cast‐iron plate, named “satsi” or “satzi,” or a flat nonstick pan after greasing it using a cloth previously dipped in a very small quantity of fat to avoid dough sticking; this is done once at the beginning of baking. The baking continues until the top surface is covered with uniformly spaced small holes, or “eyes,” similar to honeycomb, and no unbaked dough is observed. The baked langites are round, soft, spongy, and resilient, having a thickness of 3–5 mm and a diameter of 20 up to 40 cm. After baking, langites are coated or smeared with butter or margarine and consumed as is, with honey, sugar, jam, dry nuts, cheese, and similar, or after dipping in stews. Langites are prepared and consumed throughout the year as well as during some festivals and religious holidays, such as on Christ′s Ascension Day, and in some special cases, such as in the case of childbirth.

Langites show a similarity to baghrir consumed in North African countries (Algeria, Morocco, and Tunisia), which is made using semolina or flour; injera, consumed mainly in Ethiopia; lahoh or laxoox, regularly consumed in countries like Somalia, Djibouti, Kenya, Ethiopia, Yemen, and Saudi Arabia; and less similarity to uttapam, consumed in South India [4, 6]. Further, a similarity to British crumpets could be referred though this product has a much greater thickness [7]. It is important to note that, except for wheat flour or semolina, other grains like teff, teff‐pearl millet‐buckwheat, sorghum‐carrot pulp, lupine, maize, rice and chickpea, and teff‐maize‐rice are used [3, 5, 8, 9]. Baker′s yeast or sourdough is used in langites fermentation, whereas in products like injera, laxoox, and uttapam, mixed cultures comprising lactic acid bacteria, yeast, acetic acid bacteria, and other bacteria and wild yeasts are used. It should be noted that microflora is also dependent on the raw materials used and the use of previously prepared batter [8, 10].

Chickpea (*Cicer arietinum*) and lentil (*Lens culinaris*) are two of the most popular and nutritious legumes worldwide due to their affordability and health benefits. Both are rich in essential nutrients and functional compounds, offering comprehensive nutritional profiles by providing proteins, dietary fibers, carbohydrates, essential amino acids, oligosaccharides, minerals, vitamins, and bioactive compounds. In recent years, efforts have been made and are ongoing to incorporate chickpea and lentil flour in some bakery products. These legumes increase protein content and affect dough rheology, texture, color, and sensory properties of the products. Similar to langites products, attempts have been made to incorporate legumes in their formulation, showing that such products are improved and more acceptable, whereas some issues still remain to be solved [11–13].

So far, no research has been published on langites flat bread, though it is an important baked product with social, cultural, and nutritional importance. Therefore, the present investigation was conducted to study the effect of legume flour incorporation on the quality characteristics of traditional langites after partial flour replacement by whole‐grain red lentil and chickpea flour, thus enhancing its nutritional content. Physical characteristics of raw materials were also investigated. The quality characteristics of the final product include physicochemical, textural, and structural properties of the product, as well as sensory properties. Structural properties of the product were accessed through geometrical and morphological analysis using image analysis. Additionally, principal component analysis (PCA) was used to examine the relationships between the quality characteristics.

## 2. Materials and Methods

### 2.1. Materials

Wheat bakery flour, donated by Marra Bros Mills SA (Korinthos, Greece), was the basic ingredient used for the dough. According to the manufacturer, this flour contains 11.5% protein, 1.2% lipids, 72.4% carbohydrates, 1.1% fiber, and 0.6% ash. Two types of legumes, namely red lentils and chickpeas, were donated by Saroufim S.A. The whole grain legume flours were produced using an electric hammer mill (Magico EMC50, AMA Group, Rio Reggio Emilia, Italy). The composition of legume flours was red lentil flour 26% protein, 1.9% lipids, 42.2% carbohydrates, 12.5% fiber, and 2.7% ash; chickpea flour: 22.5% protein, 6.2% lipids, 43.8% carbohydrates, 15.1% fiber, and 3.4% ash (provided by the manufacturer). Ingredients such as sugar, salt, butter, and baker′s yeast were purchased at a local market in Athens. Wheat flour as well as legume flour (red lentil, L, and chickpea, C) were weighed, mixed, and sieved. Legume flour (red lentil and chickpea) was mixed (Chef KVC3100W, Kenwood Limited, United Kingdom) with wheat flour to obtain different wheat to legume substitutions for each type of legume, namely 100:0 (W), 90:10 (WL1 and WC1), and 70:30 (WL2 and WC2).

### 2.2. Physicochemical and Functional Properties of Flours

#### 2.2.1. Particle Size Distribution (PSD)

The PSD of the raw material (wheat and legume flours) was determined by using a digital electromagnetic sieve shaker (BA200N Compact, CISA, Barcelona, Spain) equipped with 6 sieves of various sizes, according to AACC International Method 55‐60.01 [14], with slight modifications. For the analysis, 100 g of sample was weighed and sieved for 20 min with a 2.0‐mm amplitude on a continuous cycle. The mass of the material from each sieve represented the percentage size of the flour particles, and samples were analyzed in triplicate. The median diameter (D50) was also calculated.

#### 2.2.2. Moisture Content

The moisture content of the flour samples and flour mixtures was determined by following the gravimetric method [12], where 2 g of flour samples were weighed in weighing bottles and dried in an air oven (Gallenkamp Oven BS Model OV‐160, United Kingdom) at 110°C until a constant weight was achieved. Three replications were performed for each sample.

#### 2.2.3. Water Absorption and Solubility Indices

The water absorption index of flours and flour mixtures was measured with the same method followed by Lazou and Krokida [15]. Inside a weighed glass centrifuge tube, 5 mL of distilled water and 0.2 g of flour sample were added and then agitated for 2 min on a Vortex mixer and centrifuged for 20 min at 3000 rpm. The liquid phase was transferred to a weighed bottle and dried overnight in an oven (Gallenkamp Oven BS Model OV‐160, United Kingdom) at 110°C; the remaining gel was weighed. The calculation model of WAI is

(1) WAI=mgms,

where *m*_g_ is the weight of the hydrated gel, and *m*_*s*_ is the weight of the sample.

After removing the liquid phase, the dry solids obtained were used to determine the water solubility index as

(2) WSI=mdsms100,

where *m*_*d* *s*_ is the weight of dry solids from the supernatant.

#### 2.2.4. Oil Absorption Index

The oil absorption index of flours and flour mixtures was measured using the same method followed by Lazou and Krokida [15], with a modification. In a weighed glass centrifuge tube, 3 mL of refined corn oil and 0.5 g of flour sample were added. The mixture was agitated for 1 min in a Vortex mixer and left to settle for 30 min. Then, the tube was centrifuged for 20 min at 3000 rpm. Finally, the remaining oil was drained, and the remaining gel was weighed. The calculation model of OAI is as follows:

(3) OAI=mogms,

where *m*_og_ is the weight of the oil gel.

#### 2.2.5. Bulk Density of Flours

Bulk density of flours and flour mixtures was determined by a method similar to that of Obinna‐Echem et al. [16]. A weighed 50‐mL graduated cylinder was filled with the sample by gently tapping the bottom on the laboratory bench unit, with no level diminution of the sample at the 50‐mL mark. At least three replications were performed for each flour sample. Bulk density was calculated (g/mL) as follows:

(4) ρb=weight of the sample gvolume of sample mL.

#### 2.2.6. Color Determination of Flours

To identify the color of the flour and flour mixtures, a colorimeter (HunterLab, Miniscan XE Plus, Virginia, United States) was used and measured the values of the color parameters *L* ^*^ (lightness), *a* ^*^ (green–red), and *b* ^*^ (yellow–blue), based on the CIELAB system (directional annular 45° illumination, D65 daylight mode). Color differences of the samples were calculated using the following equation:

(5) ΔΕ=L∗−L0∗2+a∗−a0∗2+b∗−b0∗2,

where $L_{0}^{*}$, $a_{0}^{*}$, and $b_{0}^{*}$ represent the sample′s values without the substitution of legume flour (100:0 substitution level). Four replicates were taken from every sample.

#### 2.2.7. Scanning Electron Microscopy

SEM was conducted for raw material samples as described by Lazou [12]. The samples were mounted on cylindrical samplers using conductive adhesive and coated with gold using a vacuum pump. The microstructure of the flour samples (wheat and legume flours) was examined with a SEM (JEOL, Model JSM‐5310, JEOL Ltd., Japan), using an accelerating potential of 20 kV.

### 2.3. Langites Production Procedure

The preparation of langites was based on the traditional procedure. For mixtures of wheat flour and legume flour at three different substitutions of 100:0, 90:10, and 70:30 (wheat flour: legume flour), the same procedure was used. The procedure includes the following steps: weighing and sieving 500 g of flour or a mixture, dissolving 25 g of yeast in a small amount of water, and adding 4 g of sugar and 6 g of salt. The flour and the rest of the water were added gradually until a total quantity of 880 g was reached. The mixture was then mixed for 9 min, and the dough was fermented for 45 min at 28°C in a climate chamber (KMF240, Binder, Tuttlingen, Germany). The baking process was performed using a hot pan (210°C) for 4 min and 30 s, where 65 g of the dough were poured circularly and evenly spread to form the product. After baking, langites were left to cool at room temperature until they were analyzed. The production procedure was conducted in three replicates.

### 2.4. Quality Characteristics Determination of Langites

#### 2.4.1. Moisture Content and Water Activity

The moisture content of the langites was measured as previously described by Lazou et al. [17]. All analyses were performed in triplicate. Two grams of each sample were placed in weighing bottles and subsequently dried in a laboratory oven at 110°C until constant weight.

The water activity (*a*_*w*_) of the langites was determined with an *a*_*w*_‐meter (Aqua Lab 4TE, Decagon Devices, Inc., United States). The results were determined by the mean values of at least three measurements.

#### 2.4.2. Texture Profile Analysis

A texture analyzer (TA.XT2i, Stable Micro Systems, United Kingdom) was used for mechanical properties determination and textural characterization of langites. The TPA test was applied in three different parts from the center of each langita. The samples were subjected to double, repeated compression to 50% deformation using a 6‐mm cylindrical probe at a speed of 1 mm s^−1^. The evaluated parameters were hardness (maximum height of the first peak), cohesiveness (area ratio during second compression to area during first compression, as a reference to elasticity), springiness (the time between the end of the first compression and the beginning of the second), and chewiness (obtained by multiplying hardness, cohesiveness, and springiness values, which expresses the energy needed to chew samples) [18].

#### 2.4.3. Color Measurement of Langites

The color parameters of the langites surface, L^*^, a^*^, and b^*^, were measured with a colorimeter (HunterLab, Miniscan XE Plus, Virginia, United States), as described in the 2.2.6 section. Color difference was calculated by using Equation (5), with $L_{0}^{*}$, $a_{0}^{*}$, and $b_{0}^{*}$ to represent the control sample (100:0 wheat: legume flour substitution). Five replicates were taken from every sample.

#### 2.4.4. Geometrical & Morphological Characterization Through Image Analysis

The top surface of the langites was used for image taking and investigation of geometrical and morphological properties of the product, which enables structural characterization. The photos were captured using a digital camera (Canon EOS 4000D, Tokyo, Japan). In addition, appropriate lighting on either side of the camera and a special lighting plate (Kaiser Slimlite Plano) were used, which reduced shadows and improved the sharpness of the photos. The images were then processed with Image‐Pro Plus v7.0.1.658 software. Through the Image‐Pro Plus, the mean diameter of each langita was measured, as well as the morphological properties of the formed “eyes” on the langita surface. More specifically, the morphological properties of the “eyes” include the number of the “eyes,” their mean diameter (mm), aspect ratio (ratio between major axis and minor axis of ellipse equivalent to object), and roundness (mm). All sample measurements were performed by following the program′s configurable macros.

#### 2.4.5. Sensory Evaluation

The sensory evaluation was carried out in accordance with Lazou et al. [12]. The method used was the quantitative descriptive analysis, according to which a panel of 12 members (5 males and 7 females) participated, consisting of students and staff from the Department of Food Science and Technology of the University of West Attica with an age range of 22–45 years old. They had been trained according to the ISO standard (ISO 13299:2016) to learn the required terminology and methods. The sensory evaluation of langites was conducted in a climate‐controlled environment, with separated booths for each panelist, using normal lighting conditions and white surfaces. The panelists were provided with white plastic cups and plates to ensure consistency throughout the process and a bottle of water in order to neutralize the aftertaste of the previous sample. The evaluation was held over 7 sessions, 2 h each, in which each panelist had to evaluate 3 or 5 dishes at a time, each containing a langita with different substitution levels of wheat to legume flour (100:0, 90:10, and 70:30). Each sample was assigned a unique three‐digit code, which was marked on the corresponding plate. The descriptive terms that were evaluated from the panelists belong to the following categories: appearance, odor, flavor, taste, texture, and overall acceptability. The panelists had to grade them using a 9‐point scale, where 1 = *lower intensity*/*not perceived feature*, 5 = *fairly perceptible feature*, and 9 = *higher intensity*/*extremely noticeable*. Table 1 summarizes the definitions of sensory attributes.

**Table 1:** Terms, attributes definitions and anchors used in the descriptive analysis of langites flatbread.

	Attribute	Definition	Anchor
Appearance	Top‐surface color	Degree of top‐surface darkness from white/yellow to brown	Light to dark
	Eye number of top‐surface	Number of eyes per cm^2^	Low to high
	Eye size	Top‐surface eye diameter	Low to high
	Eye homogeneity	Homogeneity of eye size	Low to high
	Eye distribution	Uniformity of eye distribution on sample’s surface	Low to high
Smell	Aroma	Total intensity of aroma	Low to high
Flavor	Mealy	Unbaked flour	Low to high
	Toasted and burnt	Percentage of burned samples	Low to high
	Yeasty	Level of yeasty odor	Mild to strong
Taste	Sweet	Fundamental taste sensation based on sucrose (typical)	Weak to intense
	Salty	Fundamental taste sensation based on salt (typical) (Flavor factor caused by salt)	Weak to intense
	Sour	Fundamental taste sensation caused by acids	Weak to intense
	Bitter (aftertaste)	Residual taste sensation of based on caffeine solution in water	Weak to intense
Texture by hands	Firmness	Resistance (force) to pressure by fingers	Small to large
	Elasticity (by hands)	Ability of the sample to return its original size and shape after extension	Low to high
Texture in mouth	Hardness	Force required to bite through sample	Low to high
	Springiness	Degree in which the sample returns to its original size and shape after partial compression between the lips	Low to high
	Chewiness	Number of chews required before swallowing	Low to high
	Adhesiveness	Degree to which the product adheres to the palate	Low to high
	Cohesiveness	The extent to which a material can be deformed before it ruptures	Low to high
	Hydration	Amount of saliva segregated in the oral cavity during sample chewing	Low to high

Further, hedonic sensory tests were conducted to assess general acceptance of langites. According to the experimental design [17], 50 untrained panelists—25 men and 25 women—who were graduate students and employees of the University of West Attica′s Food Science and Technology department in Athens, Greece, were also gathered for additional sample evaluation. This was done in a way that allowed for the evaluation of three replicates. The assessment was conducted in the same sensory evaluation lab, which had normal lighting and climatic control. White plates with three‐digit codes were used to display the samples. A 9‐point hedonic scale, with 1 denoting “strongly dislike” and 9 denoting “strongly like,” was used to assess the various langites preparations for general acceptance.

This study was registered and approved by the Ethics Committee in Research of the University of West Attica, under Protocol No. 6555‐23/01/2025.

### 2.5. Statistical Analysis

The effect of legume flour substitution on the functionality of flour mixtures and quality characteristics of the product was assessed through analysis of variance (ANOVA). In cases where a statistically significant effect was found, Duncan′s multiple range test was used to investigate the differences between the mean values of the parameters, with a significance level of *p* < 0.05. All the analyses were performed in IBM SPSS Statistics 29.0 software. PCA was implemented to investigate correlations between langites quality attributes and was performed with Statgraphics Centurion XV (Statgraphics, Rockville, Maryland, United States).

## 3. Results and Discussion

### 3.1. Physicochemical and Functional Properties of Flours

PSD histograms and cumulative distribution curves are presented in Figure 1A,B, respectively. Commercial wheat flour exhibited lower particle size values (D50 = 122.5 *μ*m), characterized by a narrow distribution, as it was milled commercially in a roller mill and fulfilling specifications required for baking flours. Whole grain legume flours, containing all seed components—endosperm, germ, and bran—exhibited higher particle size values. Although the two legumes were milled under the same conditions using a hammer mill, PSD curves were significantly different, demonstrating the importance of seed structure and composition for the flour derived. Lentil flour had a broader peak shifted toward larger bins (roughly 150–200 *μ*m) and a longer tail toward finer particles, reflecting higher heterogeneity. Chickpea flour presented a characteristic bimodal distribution, indicating the presence of two distinct populations of granules with different sizes: one of starch granules (50–100 *μ*m) and another of larger fiber‐rich particles (250–400 *μ*m). Overall, chickpea flour was the coarsest among the flours used, with D50 = 321.2 *μ*m. Its particle size is expected to influence hydration, gluten development, starch accessibility, and dough microstructure, though coarse chickpea particles are generally less disruptive to the gluten network than fine chickpea flour [19, 20]. Flour is considered a heterogeneous system composed of particles with different densities and morphologies [21, 22], as depicted in SEM images (Figure 1C). Wheat flour contained starch granules with characteristic shapes—lenticular for A‐type and spherical for B‐type, as previously reported [23]. Whole‐grain legume flours displayed large, irregular, aggregated particles. Lentil starch granules were oval and smooth. In lentil flour, most starch granules were embedded or fractured, forming matrices surrounded by protein [24]. Similarly, in chickpea flour images, large starch‐protein matrices were present, and starch granules were also oval‐shaped [25]. However, the SEM image revealed the coarser nature of chickpea flour, with prominent fiber fragments [12]. Coarse particles hinder proper gluten formation, reducing bubble development during fermentation. Conversely, smaller particles greatly affect flour absorption and its rheological properties [26]. In this study, chickpea flour—the coarsest sample—exhibited the highest values of WAI and WSI. WAI increased as the percentage of wheat flour was replaced with legume flour (Table 2), which is in accordance with previous findings [27]. Values for wheat‐lentil mixtures ranged from 2.07 to 2.40 g/g, whereas those for wheat‐chickpea mixtures ranged from 2.07 to 2.56 g/g. Higher water absorption values of chickpea compared with lentil flour have also been detected by Kohajdová et al. [28]. The increase in WSI was more pronounced, reflecting a greater increase of soluble components up to 432% and 397% as the proportion of lentil and chickpea flour increased from 0% to 100%, respectively. A slight increase in OAI was observed at 100% substitution with either lentil or chickpea flour. These findings confirm that composite flours with legumes have an effect on the functional properties of bakery products.

**Figure 1:**
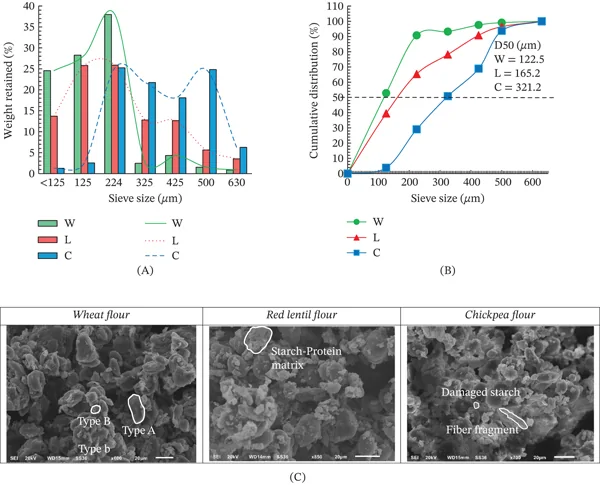
Particle size distribution of flours (W, wheat; L, lentil; C, chickpea). (A) Histogram, (B) cumulative curves, and (C) scanning electron micrographs of wheat flour, red lentil, and chickpea flour. Magnification ×600, 850, and 700, respectively.

**Table 2:** Physicochemical and functional properties of flours and their blends.

	W	WL1	WL2	L	WC1	WC2	C
Wheat:legume substitution	100:0	90:10	70:30	0:100	90:10	70:30	0:100
*Χ* % (w.b.)	13.21 ± 0.07^c^	13.39 ± 0.86^cd^	13.90 ± 0.13^d^	14.73 ± 0.65^e^	11.60 ± 0.14^b^	11.11 ± 0.13^b^	8.98 ± 0.10^a^
*ρ_b_* (g/mL)	0.75 ± 0.00^c^	0.74 ± 0.01^c^	0.81 ± 0.00^d^	0.89 ± 0.01^e^	0.70 ± 0.00^b^	0.71 ± 0.00^b^	0.64 ± 0.00^a^
L*	94.22 ± 0.23^e^	92.46 ± 0.55^d^	89.88 ± 0.19^b^	82.98 ± 0.28^a^	93.75 ± 0.24^e^	92.78 ± 0.04^d^	91.52 ± 0.13^c^
a*	1.12 ± 0.06^c^	2.33 ± 0.36^d^	5.41 ± 0.28^e^	13.52 ± 0.10^f^	−0.43 ± 0.09^a^	1.20 ± 0.16^c^	0.56 ± 0.21^b^
b*	9.04 ± 0.20^a^	11.56 ± 0.32^b^	13.61 ± 0.26^c^	25.97 ± 0.76^f^	13.60 ± 0.24^c^	14.83 ± 0.19^d^	19.51 ± 0.31^e^
*Δ* *Ε*	—	3.32 ± 0.24^a^	7.63 ± 0.10^d^	23.57 ± 0.41^f^	4.85 ± 0.24^b^	5.97 ± 0.19^c^	10.93 ± 0.34^d^
WAI (g/g)	2.07 ± 0.02^a^	2.12 ± 0.00^b^	2.12 ± 0.01^b^	2.40 ± 0.01^d^	2.11 ± 0.01^b^	2.21 ± 0.02^c^	2.56 ± 0.01^e^
WSI (g/100g)	4.96 ± 0.85^a^	6.71 ± 0.29^a^	9.83 ± 1.90^b^	26.39 ± 0.08^c^	5.40 ± 0.07^a^	9.07 ± 1.66^b^	24.66 ± 0.34^c^
OAI (mL/g)	1.66 ± 0.01^ab^	1.66 ± 0.01^ab^	1.66 ± 0.01^ab^	1.67 ± 0.01^ab^	1.63 ± 0.06^a^	1.70 ± 0.040^bc^	1.74 ± 0.01^c^

*Note:* Mean values ± standard deviation shown. Mean values in rows with a different superscript letter were significantly different as determined with the Duncan′s test (*p* < 0.05). W, wheat flour; WL, wheat‐lentil flour; WC, wheat‐chickpea flour; C, chickpea flour.

Chickpea flour presented significantly lower values of bulk density compared with wheat and lentil flour (Table 2), following the results of Nkurikiye et al. [29]. In general, finer particles present higher values of bulk density as there is lower interstitial space.

The substitution of legume flours into wheat flour significantly affected the color attributes of the blends (Table 2). For lentil flour, a marked decrease in lightness (*L* ^*^) and significant increases in redness (*a* ^*^) and yellowness (*b* ^*^) were observed, with *Δ* *E* reaching 23.57 at 100% substitution, indicating a substantial visual difference from wheat flour. Chickpea flour blends exhibited relatively smaller changes in *a* ^*^ and *b* ^*^ values, still resulting in noticeable changes (*Δ* *E* = 10.93), which could influence consumer acceptance and product development.

### 3.2. Quality Characteristics of Langites

Moisture content of langites increased significantly with higher legume flour substitution in both cases, lentil and chickpea (Table 3). As referred to 3.1. session, legume flours have higher water absorption capacity than wheat flour, probably due to their composition with higher fiber and protein content, components which bind water [30]. Water activity was high in all cases (≥ 0.986) with a slight decrease as the legume flour was increasing, especially in langites with chickpea.

**Table 3:** Physiochemical, geometrical, morphological, and textural properties of langites with different wheat to legume flour substitutions.

	W	WL1	WL2	WC1	WC2
*Wheat:legume substitution*	**100:0**	**90:10**	**70:30**	**90:10**	**70:30**
Physicochemical properties					
*X* % (w.b.)	57.60 ± 0.71^a^	59.39 ± 1.23^b^	62.07 ± 1.54^c^	58.93 ± 1.06^b^	61.82 ± 0.78^c^
*a*_*w*_	0.993 ± 0.002^c^	0.993 ± 0.001^c^	0.990 ± 0.004^b^	0.986 ± 0.003^a^	0.986 ± 0.003^a^
L^*^	75.88 ± 0.48^d^	72.78 ± 0.61^b^	68.92 ± 0.54^a^	75.33 ± 0.63^d^	74.57 ± 0.83^c^
a^*^	3.42 ± 0.12^a^	7.45 ± 0.50^d^	12.23 ± 0.23^e^	4.59 ± 0.37^b^	6.90 ± 0.25^c^
b^*^	23.79 ± 1.10^a^	24.13 ± 0.94^a^	25.92 ± 0.62^b^	23.45 ± 1.22^a^	25.57 ± 0.74^b^
*Δ* *Ε*	—	4.73 ± 0.63^b^	11.52 ± 0.38^c^	2.35 ± 0.32^a^	4.09 ± 0.38^b^
Geometrical and morphological properties					
Diameter (mm)	136.00 ± 9.10^a^	137.25 ± 4.14^a^	135.75 ± 2.75^a^	136.38 ± 1.85^a^	136.39 ± 0.00^a^
“Eye” aspect ratio	1.606 ± 0.03^a^	1.517 ± 0.05^a^	2.077 ± 0.24^b^	1.51 ± 0.05^a^	1.621 ± 0.12^a^
“Eye” roundness	1.608 ± 0.04^a^	1.707 ± 0.06^a^	3.383 ± 0.24^c^	1.792 ± 0.07^a^	2.291 ± 0.11^b^
“Eyes” diameter (mm)	0.974 ± 0.08^a^	1.115 ± 0.047^b^	1.287 ± 0.12^c^	1.031 ± 0.07^ab^	1.141 ± 0.03^b^
Number of “eyes”	1329 ± 48.8^d^	429 ± 15.11^b^	167 ± 17.23^a^	908 ± 111.95^c^	211 ± 13.86^a^
Textural properties					
Hardness (*Ν*)	0.79 ± 0.09^a^	1.35 ± 0.15^c^	1.84 ± 0.42^d^	0.99 ± 0.12^b^	1.12 ± 0.07^b^
Cohesiveness (ratio)	0.85 ± 0.01^b^	0.83 ± 0.02^b^	0.80 ± 0.02^a^	0.85 ± 0.02^b^	0.83 ± 0.02^b^
Springiness (mm)	1.001 ± 0.004^a^	1.001 ± 0.002^a^	1.002 ± 0.01^a^	0.999 ± 0.001^a^	0.999 ± 0.001^a^
Gumminess (*Ν*)	0.64 ± 0.05^a^	0.97 ± 0.04^b^	1.45 ± 0.11^c^	0.81 ± 0.02^b^	0.90 ± 0.03^b^
Chewiness (N*mm)	0.63 ± 0.06^a^	0.97 ± 0.04^b^	1.45 ± 0.11^c^	0.81 ± 0.01^b^	0.90 ± 0.04^b^

*Note:* Mean values ± standard deviation shown. Mean values in rows with a different superscript letter were significantly different as determined with the Duncan′s test (*p* < 0.05). W, wheat flour; WL, wheat‐lentil mix flour; WC, wheat‐chickpea mix flour.

Abbreviations: mm, millimeter; N, newton.

The color of langites changed significantly as legume flour was added, with red lentil addition exerting the most pronounced effect (Table 3). This can be detected also by human vision, as in almost all cases, *Δ* *Ε* was higher than 3. The only exception was the sample with 10% chickpea substitution with *Δ* *Ε* = 2.35. Lightness decreased with legume flour addition, but chickpea flour had a milder effect. Lentil flour caused a much stronger increase in redness (*a* ^*^) than chickpea, whereas yellowness (*b* ^*^) increase was the same in both cases. Furthermore, color differences might be attributed to the elevated baking temperature and the browning reactions taking place [12].

Image analysis revealed significant insights regarding morphological and geometrical properties of langites (Table 3). The diameter of langites did not change as legume flour was increased. As the roundness value moved away from 1, the eyes of langites became increasingly less round. The sample with 30% lentil flour had the most elongated eyes, as it was also confirmed from aspect ratio values. An aspect ratio of 1 means the object is perfectly circular. Figure 2B depicts the alternations in “eyes” morphology. Legume flours contain more fiber, proteins, and coarser starch granules and thus can disrupt gluten networks, decreasing the gluten strength [31]. As a result, the bubbles of gas produced during fermentation cannot be effectively entrapped and retained. In addition, there is an impact on the surface tension of the dispersion system (fermented batter) [32], favoring the breakdown processes and leading to less uniform shapes and increased surface roughness. An increased number of eyes with uniform diameter is a desirable characteristic for langites, similarly to injera, where eye distribution is also a crucial quality characteristic [8]. A sharp decrease in eye number with an increase of legume flour was detected, indicating a weaker gluten network with reduced gas retention. It should be noted that wheat dough gluten undergoes dilution as a result of chickpea or red lentil flour incorporation into wheat dough, as legume proteins replace part of the gluten‐forming wheat proteins which cannot form the same continuous, viscoelastic network [19, 20]. Two competing mechanisms might contribute to bubble stabilization enhancement: gluten dilution, leading to a reduction of structural elasticity, and increased batter viscosity. Hence, at low to moderate substitution levels, the viscosity‐enhancing effects often compensate for reduced gluten functionality, whereas at high substitution levels, the weakened protein network can no longer adequately support gas expansion. This results in a reduced number of eyes and a denser structure. Further, chickpea flour exhibited a bimodal PSD, revealing that a significant amount of both fine and coarse chickpea particles are present, which show different behavior. Fine chickpea particles, being numerous and widely distributed, can become interspersed along gluten network strands, producing widespread microscopic discontinuities. Coarse particles create larger but more localized interruptions. In this case, gluten may form a continuous matrix around or partly enveloping these inclusions [20]. Eye number decrease was clearly visible to the naked eye (Figure 2B). Figure 2A presents the relative frequency of langites eye diameter. Eye diameter increased from 0.9744 (100% wheat) to 1.287 and 1.141 mm at 30% substitution with lentil and chickpea flour, respectively. Furthermore, the control and 90:10 chickpea‐containing langites′ frequency curves are highly comparable, suggesting that their morphological features are almost similar. This suggests that both samples may share a structure and be more palatable and acceptable.

**Figure 2:**
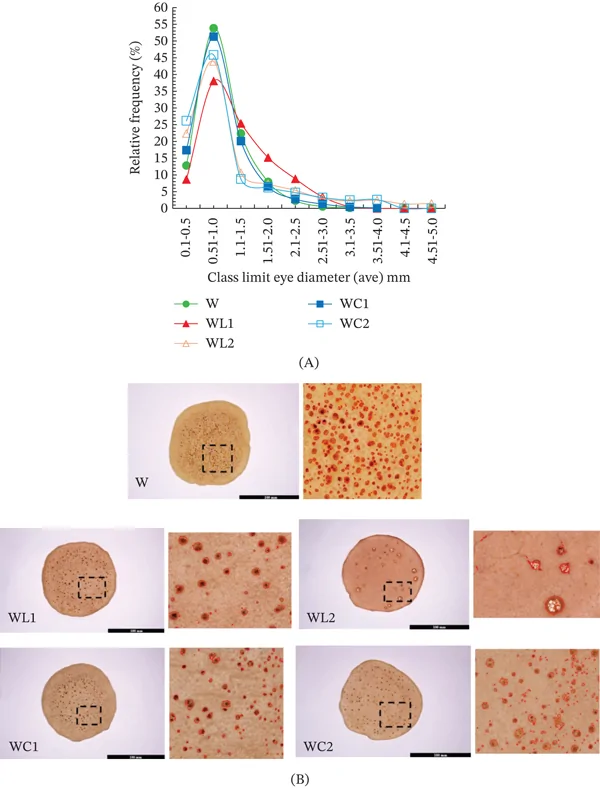
(A) Relative frequency of langites eyes diameter and (B) indicative photographs of langites and details of their eyes using image analysis software. Langites with different wheat substitution levels: 100:0 (W), 90:10 lentil or chickpea flour (WL1 or WC1), and 70:30 lentil or chickpea flour (WL2 or WC2, respectively).

Textural properties of langites were significantly influenced by both legume type and substitution level. Higher hardness values were recorded for the legume‐enriched langites, with lentil flour substitution resulting in a more pronounced increase in hardness than chickpea flour substitution at the same level. Lentil substitution at 30% resulted in the highest hardness, gumminess, and chewiness (from 0.79 to 1.84 N, from 0.64 to 1.45 N, and from 0.63 to 0.81, respectively) and the lowest cohesiveness (from 0.85 to 0.80) of the langites. Springiness was not affected by legume flour substitution. The greater increase in hardness in red lentil blends may be attributed to their higher fiber and protein content, which disrupts the gluten network, creating a denser and less elastic crumb, especially with higher levels. This may also be due to a finer particle size, which enhances water absorption and contributes to a more compact structure [33]. The instrumental hardness and chewiness of the chickpea‐based langites at the 90:10 substitution level were marginally higher than those of the control sample, suggesting that this substitution level did not significantly alter the dough′s matrix formation, leading to a product with a very similar structure and textural qualities.

Similarly, the sensory panel identified differences related to texture characteristics, firmness, springiness, and cohesiveness, which were more pronounced at the 30% substitution level (WL2 and WC2) (Figure 3A). As more legume flour was substituted, the product′s hardness, both by hand and at the first bite, increased. Characteristics of langites eyes, like number, homogeneity, and distribution, deteriorated as the substitution level increased. At 30% substitution, samples presented significantly reduced values of the aforementioned characteristics, as confirmed by their morphological characterization. Moreover, the increase in legume flour substitution had a negative impact on the product′s taste, increasing the mealy flavor, which is a characteristic effect of legume‐enriched products. Overall, the sensory profile of samples changed because of legume type and their substitution level. Control sample (W) exhibited the most desirable sensory characteristics; low‐level substitution (10%) preserved most desirable sensory properties, whereas higher substitution (30%) significantly compromised both structural texture and flavor balance.

**Figure 3:**
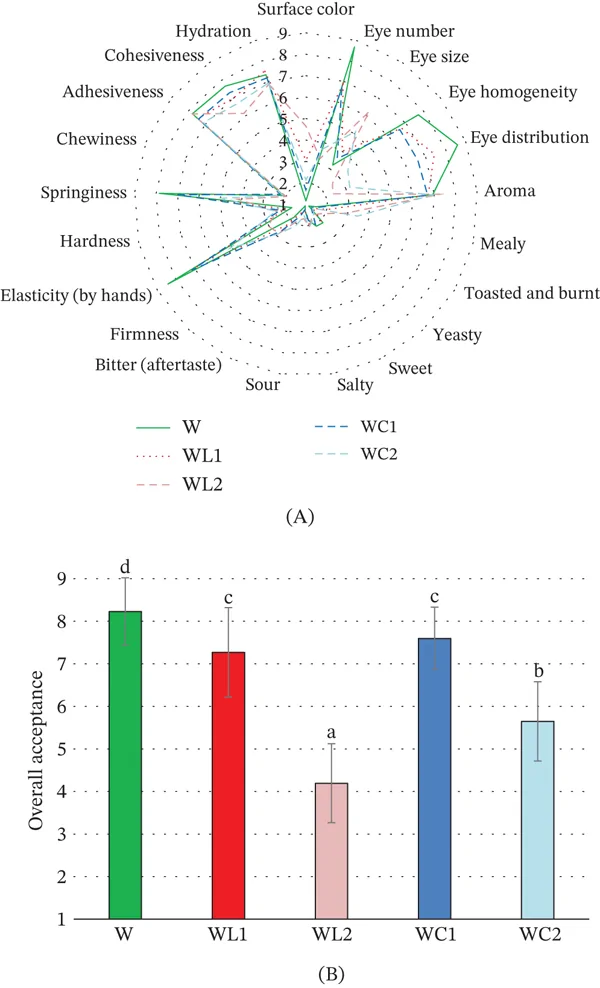
(A) Sensory profile and (B) overall acceptance of langites with different wheat substitution level: 100:0 (W), 90:10 lentil or chickpea flour (WL1 or WC1), and 70:30 lentil or chickpea flour (WL2 or WC2, respectively).

The sensory panel showed a preference for langites with the lowest legume flour content, W = 8.21 (Figure 3B). However, the wheat substitution level at 10% maintained a good acceptability, which did not differentiate significantly due to the different legume types (WL1 = 7.26 and WC1 = 7.59). A 30% substitution level resulted in a marked decline in acceptance, particularly for lentil flour (WL2 = 4.19). Similar trends have been reported in different bakery products, where legume substitution above 5%–10% significantly reduced consumer acceptability [34]. Substitution of 10% legume flour in baked rolls resulted in the most acceptable sample according to sensory evaluation [28].

The PCA biplot explained 88.55% of the total variance (Figure 4). The first component accounted for 74.51% of the variability in the initial data. It was associated mainly with texture attributes, eye characteristics, and overall acceptance, whereas the second component (13.74%) was characterized by sensory attributes and adhesiveness. Increasing firmness, moisture, and bitterness were associated with a deterioration of structural and sensory quality. The analysis indicated a positive correlation among morphological characteristics, textural properties (both instrumental and sensorial), and overall acceptability of langites. Moreover, the overall acceptability positively correlated with the number of eyes and sweet taste, indicating that a high number of eyes is crucial for the consumer and a porous structure enhances the sweetness perception. Though current evidence shows an eye number and sweet taste relationship is not directly linked by a cause‐and‐effect relationship, sweetness, and eye number are both consequences of the fermentation dynamics [35]. Starch hydrolysis generates fermentable sugars, and then carbon dioxide is responsible for eye formation. However, the portion of sugars produced remains, contributing to mild sweetness, as langites′ fermentation takes place using only baker′s yeast and no lactic acid bacteria. Therefore, products exhibiting a high number of small, uniformly distributed eyes frequently receive higher sweetness scores because the improved porous structure provides an enhanced flavor release during mastication, giving the impression of higher sweetness. A similar association has been reported by Hamid et al. [36], who found that in Sudanese kisra consumer sensory analysis, sweetness, porousness, smoothness, and lightness clustered together are positive drivers of product acceptance. Hence, sweetness and number of eyes (pores) were positively associated in multivariate sensory analysis, suggesting that consumers tend to perceive well‐aerated products as simultaneously sweeter and more palatable. Despite this, one cannot imply that sweetness creates more pores. The uniform distribution of the eyes of the products results in a honeycomb‐type aerated open structure, which corresponds to increased elasticity, brightness, and sweetness of the product, and consequently results in increased acceptability. Overall acceptance was negatively correlated with hardness, chewiness, color, eye size, and aspect ratio of langites. The formulations containing a 90:10 wheat‐to‐legume substitution presented similar properties to the control, and for this reason, had higher overall acceptance than the products with a 70:30 wheat‐to‐legume substitution. The latter had larger, unevenly distributed pores with increased hardness and low elasticity. The addition of legume flour acts negatively on the development of the gluten network, combined with the higher fiber content due to whole wheat flour. Similar results have been found for other porous products containing legume flour, such as extruded products, biscuits, bread, etc. [12, 34].

**Figure 4:**
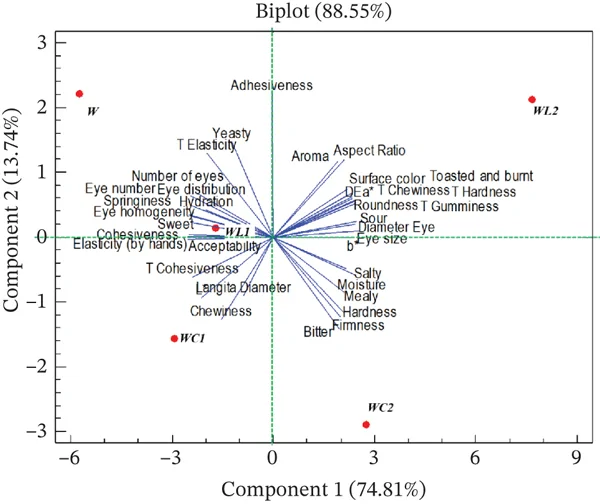
Principal component analysis of langites characteristics with different wheat substitution levels: 100:0 (W), 90:10 lentil or chickpea flour (WL1 or WC1), and 70:30 lentil or chickpea flour (WL2 or WC2, respectively).

## 4. Conclusions

In the present study, langites, a traditional fermented flatbread, were produced by substituting wheat flour with whole‐grain red lentil or chickpea flour at different levels of wheat‐to‐legume flour (100:0, 90:10, and 70:30). The analysis of flour properties revealed that whole‐grain legume flours displayed large, irregular, aggregated particles. Legume starch granules were oval and smooth. Chickpea flour exhibited the coarser particle, influencing the functional properties of the mixture. The substitution of legume flour in langites formulations significantly influenced the product quality characteristics. The samples′ color changed significantly, and this change was particularly noticeable in red lentil formulations, being visible to the unaided eye (*Δ* *E* > 3) for all substitutions. Morphological and textural characterization revealed important structural characteristics of langites. Image analysis showed that the increase in legume flour substitution decreased the number of eyes and increased their mean diameter and aspect ratio. The 90:10 chickpea flour substitution exhibited similar eye morphology and eye size distribution. The hardness of langites, both instrumental and sensorial, increased with the substitution of legume flour in the langites formulation, whereas the elasticity was the same. Important correlations between morphological, textural, and sensorial characteristics were found. The overall acceptability positively correlated with the number of eyes and sweet taste and negatively with hardness, color, and increased eye diameter. Regardless of the type of legume, a low substitution level (90:10) effectively maintained desirable sensory and structural characteristics with good consumer acceptance, whereas a higher substitution level (70:30) seriously lowered product quality, especially in lentil‐enriched samples.

## Author Contributions

A.E.L.: conceptualization, methodology, investigation, data curation, formal analysis, validation, writing—original draft, writing—review and editing, and supervision. E.D.G.: methodology, investigation, formal analysis, validation, and writing—original draft. D‐M.B.: investigation, methodology, formal analysis, validation, and writing—original draft. E‐M.T.: investigation, methodology, formal analysis, validation, and writing—original draft. S.P.: investigation, methodology, data curation, validation, writing—original draft, and writing—review and editing.

## Funding

No funding was received for this work.

## Disclosure

All authors read and approved the final version of the manuscript.

## Ethics Statement

The Ethics Committee in Research of the University of West Attica, Athens, Greece, approved the study protocol with Reference Number 6555‐23/01/2025. The experimental design was conducted in accordance with the applicable laws and regulations for humans. Written informed consent was obtained from all participants prior to their participation. Participants were informed about the purpose of the study, the procedures involved, the voluntary nature of participation, and their right to withdraw at any time without penalty. Participant confidentiality and anonymity were maintained throughout the study.

## Conflicts of Interest

The authors declare no conflicts of interest.

## Data Availability

The data that support the findings of this study are available from the corresponding author upon reasonable request.
